# Thoracic Segmental Spinal Anesthesia for Laparoscopic Surgeries in Pediatric Patients: A Retrospective Case Series

**DOI:** 10.7759/cureus.81320

**Published:** 2025-03-27

**Authors:** Naresh Paliwal, Imran Ahmed Khan, Rajeev K Shahi

**Affiliations:** 1 Anesthesiology, Dr. Panjabrao Deshmukh Memorial Medical College, Amravati, IND; 2 Community Medicine, KMC Medical College and Hospital, Maharajganj, IND; 3 Surgery, Autonomous State Medical College, Kushinagar, Kushinagar, IND

**Keywords:** dexket sedation, hemodynamics, pediatric surgery, regional anesthesia, thoracic segmental spinal anesthesia

## Abstract

Thoracic segmental spinal anesthesia (TSSA) is a developing regional anesthesia approach that involves administering local anesthetics into the thoracic spine to produce a targeted nerve block. This segmental block provides stable hemodynamics and early recovery. Lumbar spinal anesthesia is a common practice in children, and TSSA can be a promising technique due to its advantages. Despite the theoretical advantages of TSSA in pediatric patients, there is a paucity of literature describing its clinical use, safety profile, and outcomes in this population. This case series presents our initial clinical experience with TSSA in pediatric patients undergoing various laparoscopic surgical procedures. We aim to highlight the feasibility and safety of TSSA in the pediatric population while contributing to filling the gap in the literature supporting its broader application. Five pediatric patients undergoing various surgeries under TSSA were included in this study conducted between July 2021 and April 2022 at a single center. Demographic, clinical, TSSA technique, intraoperative, and postoperative data were extracted from hospital records. There were three male and two female patients between the ages of 6 and 13 years. One patient had sickle cell anemia, and one was suffering from an upper respiratory tract infection. All cases were conducted successfully under TSSA without significant complications. This study demonstrates that TSSA can be a feasible and safe regional anesthetic technique in pediatric patients undergoing surgeries confined to thoracic and upper abdominal dermatomes. While our findings are encouraging, larger prospective studies are necessary to establish standardized protocols, optimize dosing strategies, and further validate the role of TSSA in pediatric anesthesia practice.

## Introduction

Thoracic segmental spinal anesthesia (TSSA) is an evolving regional anesthesia technique that involves intrathecal administration of local anesthetics (LAs) at thoracic spinal levels to achieve segment-specific blockade [1]. While traditionally considered a high-risk approach due to concerns regarding potential spinal cord injury, recent studies have demonstrated its safety and efficacy when performed with proper anatomical understanding and correct technique [2,3]. There is plenty of literature available regarding TSSA use in adult patients across various surgical specialties [4,5]. TSSA offers advantages such as rapid onset, effective pain relief, hemodynamic stability, and reduced anesthetic drug requirements [6,7].

In contrast, its practice in pediatric anesthesia remains relatively underexplored. Pediatric patients present unique anatomical and physiological considerations that often make anesthetic management more challenging [8]. General anesthesia (GA) continues to be the mainstay in the pediatric population. However, concerns such as airway issues, delayed recovery, and long-term neurodevelopmental impact have led to increased interest in regional techniques as a safer alternative [9,10]. Spinal anesthesia (SA) at the lumbar region is well documented in pediatric patients [11]. TSSA, with its potential to provide effective segmental anesthesia with minimal systemic drug exposure, offers a promising option in pediatric patients, especially for surgeries confined to limited dermatomes.

Despite the theoretical advantages of TSSA in pediatric patients, most available data are limited to isolated case reports or extrapolated from adult studies, leaving a significant knowledge gap regarding its feasibility, technique modifications, and perioperative implications in children. This lack of evidence poses a barrier to the wider adoption of TSSA in pediatric practice. This retrospective case series presents our initial clinical experience with TSSA in pediatric patients undergoing various surgical procedures. We aim to highlight the feasibility, safety profile, and potential benefits of this technique in the pediatric population while contributing to filling the gap in the literature supporting its broader application.

## Case presentation

A retrospective study was conducted from data of pediatric patients undergoing laparoscopic surgeries at a single center between July 2021 and April 2022 under TSSA. Attendants of all patients were explained the TSSA procedure, and informed consent was obtained. All attendants agreed to allow the use of their data for publication, provided anonymity is maintained. Demographic, clinical, TSSA technique detail, and postoperative data were extracted from hospital records. All the cases were conducted by a single anesthesiologist with sufficient experience in TSSA and pediatric anesthesia. As this is a retrospective case series with anonymized data and no patient identifiers, formal ethical clearance was not required. All patients were followed for a period of two months through telephone, and no postoperative neurological, cardiac, or respiratory complications were reported during the two-month follow-up period.

The demographic and clinical characteristics of the participants are presented in Table 1. There were three male and two female patients between the ages of 6 and 13 years. One patient was a diagnosed case of sickle cell anemia, and one was suffering from an upper respiratory tract infection (URTI).

**Table 1:** Demographic and clinical characteristics of the participants URTI, upper respiratory tract infection

Case details	Case 1	Case 2	Case 3	Case 4	Case 5
Age (year)	6	12	13	8	6
Gender	Male	Female	Female	Male	Male
Weight (kg)	20	28	34	30	18.5
Concurrent illness	COVID-19 positive contact	COVID-19 positive contact	None	URTI	Sickle cell anemia
Diagnosis	Acute appendicitis	Twisted ovarian cyst	Bicornuate uterus with rudimentary right cornua	Perforated appendix	Cholelithiasis
Procedure	Laparoscopic appendicectomy	Laparoscopic untwisting and fixation	Laparoscopic excision of rudimentary horn	Laparoscopic appendicectomy	Laparoscopic cholecystectomy

During the preoperative evaluation, all patients and attendants were explained the pros and cons of the TSSA block. GA was avoided in view of the pandemic. An intravenous line of appropriate size was secured, and standard monitoring was attached and verified their function. None of them were given pre-block sedation, and all patients were given LA infiltration at the intended puncture site. Spinal puncture level was T10/12 or T11/12 interspace using a 27 G spinal needle in the lateral position. All patients were maintained on spontaneous respiration using Venturi masks with 2 L/min oxygen flow. All patients were given sedation in the form of dexmedetomidine and ketamine (dexket) injections. The detailed technique of the TSSA used in all cases is compiled in Table 2.

**Table 2:** Details of TSSA procedure among participants Dexa, dexamethasone; Dexem, dexmedetomidine; Dexket, dexmedetomidine and ketamine; G, gauze; LA, local anesthetic; MMA, multimodal analgesia; T, thoracic; TSSA, thoracic segmental spinal anesthesia

TSSA technique	Case 1	Case 2	Case 3	Case 4	Case 5
Position	Lateral	Lateral	Lateral	Lateral	Lateral
Preprocedural LA	Yes	Yes	Yes	Yes	Yes
Preprocedural sedation	None	None	None	None	None
Level	T11/12	T11/12	T10/11	T11/12	T10/11
Spinal needle	27 G	27 G	27 G	27 G	27 G
LA	Levobupivacaine, isobaric	Levobupivacaine, isobaric	Bupivacaine, isobaric	Levobupivacaine, isobaric	Levobupivacaine, isobaric
concentration	0.50%	0.50%	0.50%	0.50%	0.50%
Volume (ml)	1.1	1.3	1.5	1.3	1
Dose (mg)	5.5	6.5	7.5	6.5	5
Additive	Fentanyl 15 μg	Fentanyl 20 μg	Dexem 5 μg	Fentanyl 20 μg	Fentanyl 15 μg
Levels achieved	T3/4–L5/S1	T2/3–L5/S1	T2/–L5/S1	T2/3–L5/S1	T2–L5/S1
Intraop sedation	Dexket sedation (10 μg + 20 mg)	Dexket sedation (15 μg + 30 mg)	Dexket sedation (25 μg + 50 mg); Ketofol (20 mg + 50 mg)	Dexket sedation (25 μg + 50 mg)	Dexket sedation (10 μg + 20 mg)
Duration (minutes)	50	65	130	70	50
Postop analgesia	MMA (Dexa, paracetamol, and LA at the port site)	MMA (Dexa, diclofenac, paracetamol, and LA at the port site)	MMA (Dexa, diclofenac, paracetamol, and LA at the port site)	MMA (Dexa, diclofenac, paracetamol, and LA at the port site)	MMA (Dexa, paracetamol, and LA at the port site)

Perioperative hemodynamics are compiled in Table 3. Two patients presented with hypotension, which was easily treated with a fluid bolus and a small dose of phenylephrine. None of them developed paresthesia during needle insertion. One case was prolonged and suffered shoulder tip pain postoperatively.

**Table 3:** Perioperative hemodynamics and complications PONV, postoperative nausea and vomiting

Perioperative complication	Case 1	Case 2	Case 3	Case 4	Case 5
Paresthesia	No	No	No	No	No
Hypotension	No	Yes	Yes	No	No
Bradycardia	No	No	No	No	No
Respiratory difficulty	No	No	No	No	No
Shoulder tip pain	No	No	Yes	No	No
PONV	No	No	No	No	No

## Discussion

This case series highlights the feasibility, safety, and efficacy of TSSA in pediatric patients undergoing various surgical procedures. Our findings support the emerging role of TSSA as a viable regional anesthetic technique in carefully selected pediatric cases, particularly for surgeries confined to mid-to-upper abdominal or thoracic dermatomes.

Pediatric SA was first described in 1909 [12]. Traditionally, SA in children has been limited to the lumbar levels, primarily for lower abdominal and lower limb surgeries [13]. The thoracic approach has been largely avoided due to concerns regarding spinal cord injury and technical difficulty in locating the thoracic interspace. However, anatomical understanding, availability of fine needles, appropriate means for intraop monitoring, and ultrasound-guided techniques have helped mitigate these concerns, making thoracic neuraxial blocks practical and safer [2].

Certain anatomical peculiarities in children include a highly vascular pia mater combined with greater cardiac output leading to faster reabsorption of LA drugs. Laminae are cartilaginous in neonates and infants, so you have to be cautious with the paramedian approach. Ligaments are less dense, so loss of resistance may not be well appreciated [14]. There are also some physiological differences. Immature sympathetic systems and compensatory reductions in vagal activity, less vasoplegia, and smaller venous capacitance in lower limbs make cardiovascular changes related to SA less pronounced in children <5 years. In older children >8 years, a sympathetic block may induce bradycardia and hypotension. High levels of block reduce the outward motion of the lower rib cage, leading to paradoxical respiratory movements, but the diaphragm compensates for the loss of rib cage contribution [15].

Options such as GA, epidural anesthesia, lumbar SA, local anesthesia, or monitored anesthesia with sedation have been used to perform pediatric cases. While GA offers advantages for surgical control and patient comfort, it carries increased risks of cardiovascular, respiratory, and renal complications due to anatomic and physiological peculiarities. Lumbar SA would cause sympathetic block leading to venodilation and decreased preload; in TSSA, a large portion of the body experiences no venodilation, so it safeguards intraoperative adverse changes in blood pressure [16]. Anatomically, it is shown that thoracic nerve roots are thinner with lesser CSF volume compared to lumbar nerve roots; hence, a lower drug volume is required for TSSA and hence a lesser chance of LA toxicity.

TSSA was successfully implemented in all patients. The first two cases were asymptomatic primary contacts of COVID-19-positive cases. Case 4 was suffering from a URTI. GA may cause respiratory complications, and being an emergency, it was conducted under TSSA. Case 5, with sickle cell disease, was evaluated and managed by a multidisciplinary team as per standard guidelines [17]. Isobaric LA was used in all cases with fentanyl as an adjuvant except Case 3, where dexmedetomidine was used owing to the expected long duration of surgery.

All the patients in our report were given intraoperative sedation in the form of dexket to make them calm and comfortable. The combination of dexket makes pharmacological sense; they balance the hemodynamic and adverse effects of each other [18,19]. Dexmedetomidine prevents tachycardia, hypertension, salivation, and emergence from ketamine. Ketamine prevents bradycardia and hypotension caused by dexmedetomidine. Ketamine also speeds up the onset of sedation and eliminates the slow onset of dexmedetomidine. A common regimen utilized is a bolus dose of 0.5 μg/kg of dexmedetomidine + 1 mg/kg of ketamine, mixed together, diluted, and given slowly over a minute to initiate target sedation. Maintenance doses of 0.25 μg/kg dexmedetomidine + 0.5 mg/kg ketamine are given as needed.

In our case series, TSSA provided excellent intraoperative analgesia and stable hemodynamics. The segmental blockade allowed for reduced total drug dosage, potentially minimizing the risk of systemic toxicity and postoperative respiratory depression, a significant advantage in pediatric patients. Various studies have shown the feasibility and safety of TSSA for laparoscopic surgeries in adult patients [20,21]. Furthermore, early recovery and reduced exposure to GA may offer long-term neuroprotective benefits, particularly in infants and young children where anesthetic neurotoxicity remains a concern.

Importantly, no major adverse events or neurological complications were encountered, reinforcing the safety of this technique when performed with proper patient selection and meticulous technique. Our findings are in line with the limited reports available in the literature, which also demonstrate favorable outcomes with TSSA in pediatric patients [22]. However, the absence of large-scale data and standardized protocols continues to hinder widespread clinical adoption.

This retrospective case series addresses a notable gap in pediatric regional anesthesia literature by providing real-world evidence supporting the feasibility of TSSA. It also opens avenues for further exploration into its potential benefits over GA, particularly in resource-constrained settings or patients with contraindications to GA. However, patients preferred this technique, which needs to be assessed carefully and reserved for experienced clinicians with a good learning curve [23].

The study has certain limitations. The sample size is small, and the absence of a control group limits the ability to draw definitive conclusions. Additionally, longer-term follow-up was not performed. Future prospective studies with larger cohorts and randomized designs are warranted to establish standardized guidelines, dosing regimens, and indications for TSSA in pediatric practice.

## Conclusions

This study demonstrates that TSSA is a feasible and safe regional anesthetic technique in pediatric patients undergoing surgeries confined to thoracic and upper abdominal dermatomes. The technique provided excellent intraoperative analgesia and stable hemodynamics and avoided the potential risks associated with GA. While our findings are encouraging, larger prospective studies are necessary to establish standardized protocols, optimize dosing strategies, and further validate the role of TSSA in pediatric anesthesia practice. Until then, its use should be reserved for carefully selected cases and performed by experienced clinicians well versed in thoracic neuraxial techniques and pediatric anesthesia.
